# Increasing sensitivity of methane emission measurements in rice through deployment of ‘closed chambers’ at nighttime

**DOI:** 10.1371/journal.pone.0191352

**Published:** 2018-02-01

**Authors:** Reiner Wassmann, Ma. Carmelita Alberto, Agnes Tirol-Padre, Nghia Trong Hoang, Ryan Romasanta, Caesar Arloo Centeno, Bjoern Ole Sander

**Affiliations:** 1 International Rice Research Institute (IRRI), Los Baños, Philippines; 2 Karlsruhe Institute of Technology, Institute for Meteorology and Climate Research, Garmisch-Partenkirchen, Germany; 3 Hue University of Agriculture and Forestry, Hue City, Vietnam; University of Delhi, INDIA

## Abstract

This study comprises field experiments on methane emissions from rice fields conducted with an Eddy-Covariance (EC) system as well as test runs for a modified closed chamber approach based on measurements at nighttime. The EC data set covers 4 cropping seasons with highly resolved emission rates (raw data in 10 Hz frequency have been aggregated to 30-min records). The diel patterns were very pronounced in the two dry seasons with peak emissions at early afternoon and low emissions at nighttime. These diel patterns were observed at all growing stages of the dry seasons. In the two wet seasons, the diel patterns were only visible during the vegetative stages while emission rates during reproductive and ripening stages remained within a fairly steady range and did not show any diel patterns. In totality, however, the data set revealed a very strong linear relationship between nocturnal emissions (12-h periods) and the full 24-h periods resulting in an R^2^-value of 0.8419 for all data points. In the second experiment, we conducted test runs for chamber measurements at nighttime with much longer deployment times (6 h) as compared to measurements at daylight (typically for 30 min). Conducting chamber measurements at nighttime excluded drastic changes of temperatures and CO_2_ concentrations. The data also shows that increases in CH_4_ concentrations remained on linear trajectory over a 6h period at night. While end CH_4_ concentrations were consistently >3.5 ppm, this long-term enclosure represents a very robust approach to quantify emissions as compared to assessing short-term concentration increases over time near the analytical detection limit. Finally, we have discussed the potential applications of this new approach that would allow emission measurements even when conventional (daytime) measurements will not be suitable. Nighttime chamber measurements offer an alternative to conventional (daytime) measurements if either (i) baseline emissions are at a very low level, (ii) differences of tested crop treatments or varieties are very small or (iii) the objective is to screen a large number of rice varieties for taking advantage of progress in genome sequencing.

## Introduction

Wetland rice fields are one of the main sources of CH_4_ because flooded soils provide favorable conditions for CH_4_ production and emission. This will be aggravated by an increase in rice cultivation needed to meet the growing demand for food in the coming decades. However, CH_4_ emissions during the growing season can be reduced by various crop management and irrigation practices [1, 2].

Recording GHG emissions in the field represents the most direct measure of providing empirical evidence of emission background levels and mitigation impacts. In the past, GHG emission measurements in rice have been conducted either as part of research projects or for in the context of National Communications to the UNFCCC requiring country-specific Emission Factors as part of the Tier 2 for GHG emission inventories [3]. More recently, the attention in climate policy has shifted to mitigation projects, e.g. as part of Nationally Appropriate Mitigation Actions (NAMA). The credibility of mitigation efforts will inherently depend on adequate MRV strategies (Measurement, Reporting, Verification) for quantifying GHG net-savings by a given mitigation technology.

The common approach for recording GHG emissions is closed chamber method with manual sampling [4,5]; the main advantages of this method are its low costs and flexibility. During chamber deployment, air samples are collected with a syringe and can be stored in vials before analyzing them through gas chromatography. The CH_4_ fluxes are then calculated by measuring the rates of change in CH_4_ concentrations inside the chamber for a period of about 30 minutes [6]. While these chamber measurements are typically done once a day during 0900H - 1100H, his approach has two principle shortcomings:

1. The increments in CH_4_ concentrations inside the chambers are relatively small due to the short enclosure time in the fields. Enclosure is typically limited to 30 min to avoid excessively high temperatures inside the chambers and long interruptions of photosynthesis (due to low CO_2_ level and lack of radiation in the transparent chambers). In turn, such short enclosure intervals will result in low increments in CH_4_ concentrations. Given detection limits of analytical equipment as well as natural variations (noise levels), emission rates have to be derived from recording the slope of concentrations over time. This requires a minimum of 3 samples (the recommended number is 4) for each chamber deployment.
2. High dynamics in emission rates during daytime represents a major bias for obtaining average emissions for 24-h periods. CH_4_ emissions from rice fields are correlated with dynamic changes in solar radiation and temperature [7–9]. Labor and logistic requirements pose an inherent limitation to number of measurements per day. Measurements at 0900H – 1100H are based on the assumption that emission rates in this time window will reflect the average over a 24-h cycle. It seems obvious that a single 30-min interval recorded during a period of highly dynamic emission rates in the field may substantially deviate from average values for a 24-h cycle.

As an alternative approach, we are assessing measurements conducted during nighttime that will allow a longer enclosure times without much alteration of the conditions inside the chamber. Neither temperature increase nor CO_2_ depletion will occur during chamber deployment at night. Given that emission rates are more stable during nighttime [10, 11] this approach will also allow a series of consecutive measurements without time bias.

The objectives of this paper are:

- to assess temporal patterns of CH_4_ fluxes over 24-cycles derived from high-resolution data sets obtained with an Eddy Covariance system over 4 cropping seasons,
- to evaluate the statistical relationship between average flux rates for the nocturnal period vs average flux rates for the entire 24-h cycle,
- to provide proof of concept on the reliability of nighttime chamber measurements by conducting a set of experiments with long enclosure times, and
- to assess the scope of this new approach for emission experiments for different experimental purposes and settings where measurements during daylight will be unsuitable.

## Materials and methods

### Experiment A: Eddy covariance measurements

#### Site description

The study site used is a 4 ha (200 m x 200 m) irrigated rice field located in the Zeigler Experiment Station of the International Rice Research Institute (IRRI) in Los Baños, Laguna, Philippines, (14° 8’ 27.7” N, 121° 15’ 54.98” E). The site has a slope of 1% with a northeasterly aspect, and an elevation of 27 m above sea level. The soil was characterized as Lithic Haplustept [12] varying in texture from loam to clay and overlying volcanic tuff evident at 0.3 m to 1.2 m depth. Soil analysis (0–0.15 m) showed an average pH of 6.44 (1:1 soil/water suspension), 1.42% total C, 0.13% total N, 55.7 mg kg^-1^ available P, and 1.42 cmol_(+)_ kg^-1^ exchangeable K. IRRI weather station database (1979–2012) had recorded an annual mean precipitation (± standard deviation) of 2112 ± 409 mm and an annual mean air temperature of 27.5 ± 0.33°C. The wind predominantly comes from the northeasterly direction during most parts of the year (mainly in October to May).

Rice is grown in the Philippines during two distinct seasons–the dry (DS) and wet (WS); which are climatically characterized by high solar radiation, less precipitation, and lower average temperature in the DS than in the WS [13]. The DS usually starts in December and ends in May while the WS is from June to November [14]. At the start of each season, the field was prepared by dry cultivation (disc plowing and rotavation); followed by land soaking, plowing, and harrowing which incorporated weed biomass and straw residues from the previous rice crop; and finally leveling. Using mechanical transplanter, 14–18 day-old rice seedlings of cultivar NSIC Rc222 were transplanted at a spacing of 30 cm x 16 cm. Fertilizers (14-14-14) were applied basally at 30–32 kg ha^-1^ in the DS and 23 kg ha^-1^ in the WS. Additional urea fertilizer was top dressed in two splits resulting in total N fertilizer applications of 165–167 kg N ha^-1^ in the DS and 91–103 kg N ha^-1^ in the WS. The soil was kept saturated with no standing water during the early vegetative stage from 0–7 days after transplanting (DAT) to allow the seedlings to recover from transplanting shock and to prevent damage from golden apple snails. Then the field was kept flooded with 3–5 cm depth standing water until about two weeks before harvest. The irrigation water comes from deep wells pumped into a reservoir, which has a pH: 7.83 and contains low levels of bicarbonates and nitrates (HCO_3_‾: 0.00434 mol L^-1^; NO_3_‾-N: < 0.3 mg N L^-1^).

#### EC measurements

The EC equipment used in this study and the features of the EC measurements at the IRRI experimental farm have been describes in several publications. Briefly, this method calculates mass and energy fluxes over the plant canopy. The EC system was placed at the center of the 4ha-study site, having sufficient upwind fetch of homogenous vegetation (rice) required for adequately measuring mass and energy fluxes using the EC method. Footprint analysis [15] showed that 90% of the flux measurements were recorded from within a range of approximately 100 m.

Our EC system consisted of the following sensors: (1) a sonic anemometer-thermometer (CSAT3, Campbell Scientific, Inc., USA) which measured three dimensional wind speed and sonic temperature; (2) an open-path CO_2_/H_2_O gas analyzer (LI-7500A, LI-COR Inc., Lincoln, NE, USA) which measured fluctuations in CO_2_ and water vapor densities; and (3) an open-path methane analyzer (LI-7700, LI-COR Inc., Lincoln, NE, USA) which measured CH_4_ concentrations [16].

For the meteorological measurements, net radiation and incident photosynthetically active radiation were measured at 2.79 m height using 4-component net radiometer (NR01, Hukseflux Thermal Sensors, Inc., USA) and quantum sensor (LI-190S, LI-COR Inc., Lincoln, NE, USA), respectively. Our system also included air temperature and relative humidity probes (HMP45C, Campbell Scientific, Inc., USA) installed at 2.05 m height; three Type T thermocouples (Omega Engineering Inc., USA) used to measure floodwater temperature at 2.5 cm above the soil, and soil temperatures at 2.5 and 5 cm soil depths; water content reflectometer (CS616, CSAT3, Campbell Scientific, Inc., USA), which measured soil volumetric water content (VWC); and two soil heat flux plates (HFP01, Hukseflux Thermal Sensors, Inc., USA) installed at 5 cm soil depth to measure soil heat flux at 5 cm depth. Soil heat flux is the sum of the soil heat flux measured at 5 cm soil depth, heat storage in the top 5 cm soil, and heat storage in the floodwater.

#### Determination of diel patterns in CH_4_ fluxes

The terminology used in this paper defines a ‘diel’ interval for one 24-h cycle with data points from 00:30H to 24:00 that are broken up into ‘nocturnal’ and ‘diurnal’ records. To avoid confusion, we do not use the term ‘daily emission rates’ in this study. Daily refers to an event occurring every day and not to an interval. However, we acknowledge that what we call ‘diel emission rates’ corresponds to ‘daily emission rates’ as used in other studies that do not distinguish between specific periods within a 24-h cycle. The nocturnal records comprise the data points from 0030H to 0600H and 1830H to 2400H of a given day while diurnal records cover from 0630H to1800H. The term ‘nighttime’ is more loosely defined denoting individual data points or shorter intervals, e.g. for the chamber measurements. To determine the diel patterns of CH_4_ fluxes and its driving factors, all quality-controlled data (not gap-filled) were partitioned into 3 sub-groups according to plant growth stages (vegetative, reproductive, and ripening). The data in each sub-group were binned by time of day and then averaged for all days during the measurement period. Table 1 shows the actual dates encompassing the different growth stages for 2013 and 2014 dry (DS) and wet (WS) seasons.

#### Statistical analysis

Pearson correlation analysis and simple linear regression analysis were done using PROC MIXED in SAS® version 9.3 [17].

**Table 1:** Actual dates encompassing the different growth stages in 2013DS, 2013WS, 2014DS, and 2014WS (DS–dry season; WS–wet season).

	2013DS		2013WS		2014DS		2014WS	
Growth stage	Date	No. of days	Date	No. of days	Date	No. of days	Date	No. of days
Vegetative	20 Dec 2012–27 Jan 2013	39	27 June– 7 Aug 2013	42	2 Dec 2013 – 9 Jan 2014	39	17 June– 28 July 2014	42
Reproductive	28 Jan– 3 Mar 2013	35	8 Aug– 11 Sept 2013	35	10 Jan– 13 Feb 2014	35	29 July– 1 Sept 2014	35
Ripening	4 Mar– 2 Apr 2013	30	12 Sept– 22 Oct 2013	41	14 Feb– 15 Mar 2014	30	2 Sept– 1 Oct 2014	30
Total		104		118		104		107

### Experiment B: Nighttime closed-chamber measurements

#### Closed chamber field sampling

The closed-chamber measurements were conducted at the Zeigler Experiment Station of the International Rice Research Institute (IRRI) in 2 nights (74 and 81 DAT) of the DS 2017. A plastic base with a diameter of 50 cm was inserted about 10 cm into the soil at a location representing average plant density of a given plot. These bases were installed at least a day before sample collection. The base height and water depth inside the frames were measured at each gas sampling time. The gas collection chambers, fabricated from a plastic pail with a height of 70 cm (120L volume) were equipped with a thermometer and a sampling port; and a battery-operated fan was installed inside. The EIUK RASI 700 multi gas analyzer was also connected to the chamber through a small diameter tubing to measure the oxygen concentration. At the time of sampling, the gas collection chambers were placed on the trough of the bases and sealed with water.

The CH_4_ fluxes were measured between 18:00H and 24:00H from plots with two different irrigation treatments, namely (i) continuous flooding and (ii) alternate wetting and drying. Those treatments were chosen to generate high and low background levels of methane emissions, respectively. For each treatment, three chambers were installed for gas collection. Gas samples were collected from each of the six chambers at 0, 15, 30, 60, 180 and 360 mins after chamber deployment.

#### Lab analysis and calculation of flux rates

Gas samples (50 ml) were stored under pressure in 30 ml evacuated glass vials until analysis using an SRI 8610C gas chromatograph (GC) equipped with a flame ionization detector for analysis of CH_4_. The GC was calibrated against 6 concentrations (2, 5, 10, 20 50, and 100 ppm) of CH_4_ calibration gas from Matheson Tri•Gas, which showed a linear increase in peak area. Four ml of each gas sample was injected into the GC for analysis.

According to the MIRSA guidelines [5], the emission rate is termed as ‘*Flux*_*CH4*_’ (mg CH4 m^-2^ h^-1^) in the following equation:

$${Flux}_{CH4}=\frac{\Delta C}{\Delta t}\times \frac{V}{A}\times \rho \times \frac{T_{0}}{T_{0}+T}$$ (Eq 1)

where

$\frac{\Delta C}{\Delta t}$ = concentration change over time (ppm-CH4 h^-1^)
$\frac{V}{A}$ = Chamber volume over area (m) corresponding to chamber height in regularly shaped chambers
*ρ* = gas density (0.717 kg m^-3^)
*T*_*0*_ = standard temperature (0°C = 273°K) and
*T* = mean chamber temperature (°C)

#### Determination of Limit of Detection for GC analysis and fluxes

Limit of Detection (LOD) is defined as “the lowest concentration level that can be determined to be statistically different from a blank (99% confidence)” [18]. In the context of emission studies based on GC analysis, this value is typically calculated by

$${LOD}_{gc}=3\times SD$$ (Eq 2)

where SD stands for standard deviation of the measured ambient air concentrations [5]. The specific LOD_gc_ value of our set-up was determined from repeated analyses of 10–20 samples of ambient air stored in the same gas containers that are used for gas sampling. One example for a repeated analyses of CH4 in ambient air with our GC set up is shown in the MIRSA guidelines [5] (see page 54/ Case 1) resulting in SD = 0.1 and a subsequent LOD_gc_ = 0.3.

The LOD_gc_ translates into a Limit of Detection for CH_4_ flux measurement (LOD_flux_) through the following equation:

$${LOD}_{flux}={LOD}_{gc}\times \frac{V}{A}\times \rho \times \frac{T_{0}}{T_{0}+T}\times \frac{1}{t}$$ (Eq 3)

where (in addition to acronyms defined above) *t* corresponds to the deployment time of the chamber (in hours). From this equation, it is understood that LOD_flux_ can be decreased by increasing the deployment time of the chamber—which is tantamount to increasing sensitivity of the flux measurement.

## Results

### Experiment A: Eddy covariance measurements

#### Diel variations in CH_4_ fluxes and correlation with radiation and temperature

**Fig 1:**
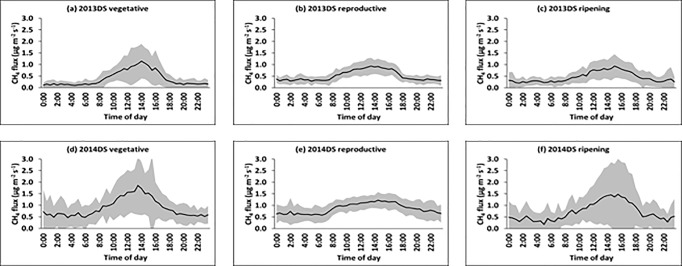
a,b,c,d,e,f. Mean diel patterns of CH_4_ flux during the different growth periods of the rice plant (vegetative, reproductive, and ripening) in the dry seasons (DS) of 2013 (a,b,c) and 2014 (d,e,f). The solid line represents the mean of all days at a given time of day (in 30 min intervals) and the shaded area represents the standard deviation.

**Fig 2:**
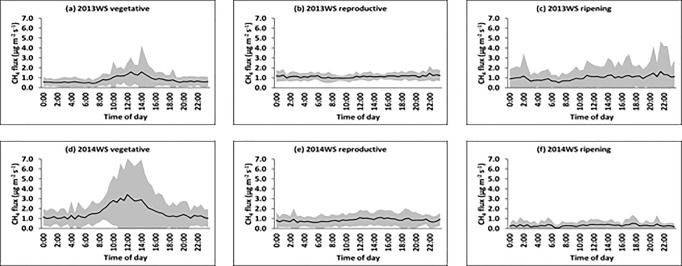
a,b,c,d,e,f. Mean diel patterns of CH_4_ flux during the different growth periods of the rice plant (vegetative, reproductive, and ripening) in the wet seasons (WS) of 2013 (a,b,c) and 2014 (d,e,f). The solid line represents the mean of all days at a given time of day (in 30 min intervals) and the shaded area represents the standard deviation.

The available data set covers two dry and two wet seasons. Dry and wet seasons showed very distinctive flux patterns as can be seen in Figs 1 and 2, so that we present them here separately. All seasons are divided into three growth stages, namely vegetative (typically from day 0 to 43 days after transplanting, DAT), reproductive (44 to 79 DAT) and ripening stage (80 to 110 DAT). During the dry seasons (Fig 1), all growth stages displayed distinct diel patterns in CH_4_ fluxes. The amplitude of the diel CH_4_ flux was higher during the vegetative compared to the reproductive and ripening growth stages. Throughout the season, CH_4_ fluxes were very low from 0000-0630H and started to increase at around 0700H - 0830H, reached a peak at around 1330H - 1530H, and then decreased to low values again after 1900H.

The diel fluxes of CH_4_ had significant and very strong positive relationship with solar radiation and temperatures, namely air temperature (*Ta*), floodwater temperature at 2.5 cm above the soil (*T*_*fw*_ *2*.*5 cm*), soil temperature at 2.5 cm depth (*T*_*s*_ *2*.*5 cm*), and soil temperature at 5 cm depth (*T*_*s*_ *5 cm*). As can be seen in Table 2, the correlations persisted throughout the different growth stages of the rice plants in both dry seasons resulting in *r* values ranging from 0.74 to 0.97.

**Table 2:** Pearson correlation analysis between the diel fluxes of CH_4_ and air temperature (*Ta*), floodwater temperature at 2.5 cm above the soil (*T*_*fw*_ *2*.*5 cm*), soil temperature at 2.5 cm depth (*T*_*s*_ *2*.*5 cm*), soil temperature at 5 cm depth (*T*_*s*_ *5 cm*), and solar radiation (*SR*) during the different growth stages of the rice plants (vegetative, reproductive, and ripening) in 2013 and 2014 dry seasons (DS).

	2013DS						2014DS					
	vegetative		reproductive		ripening		vegetative		reproductive		ripening	
Variables	*r*	*ρ*	*r*	*ρ*	*r*	*ρ*	*r*	*ρ*	*r*	*ρ*	*r*	*ρ*
*Ta*	0.95	<0.0001	0.96	<0.0001	0.91	<0.0001	0.94	<0.0001	0.97	<0.0001	0.93	<0.0001
*T*_*fw*_ *2*.*5 cm*	0.97	<0.0001	0.96	<0.0001	0.89	<0.0001	0.86	<0.0001	0.91	<0.0001	0.87	<0.0001
*T*_*s*_ *2*.*5 cm*	0.95	<0.0001	0.91	<0.0001	0.95	<0.0001	0.95	<0.0001	0.94	<0.0001	0.96	<0.0001
*T*_*s*_ *5 cm*	0.88	<0.0001	0.76	<0.0001	0.92	<0.0001	0.92	<0.0001	0.90	<0.0001	0.93	<0.0001
*SR*	0.86	<0.0001	0.88	<0.0001	0.74	<0.0001	0.87	<0.0001	0.78	<0.0001	0.79	<0.0001

During the wet seasons in 2013 and 2014 (Fig 2), distinct diel pattern in CH_4_ fluxes was only observed during the vegetative stage of the rice plant. This could most probably be due to unstable weather patterns including heavy monsoon rains and occasional typhoons that are characteristic for the period of August to October in the Philippines when the plants pass through the reproductive and ripening stages of the wet season crop [19].

Consistently, the diel fluxes of CH_4_ had significant and very strong positive relationship with *Ta*, *T*_*fw*_ *2*.*5 cm*, *T*_*s*_ *2*.*5 cm*, *T*_*s*_ *5 cm* and *SR* during the vegetative growth stage of the rice plants in 2013 and 2014 wet seasons. Pearson correlation analysis showed *r* values ranged from 0.77 to 0.95 (Table 3).

**Table 3:** Pearson correlation analysis between the diel fluxes of CH_4_ and air temperature (*Ta*), floodwater temperature at 2.5 cm above the soil (*T*_*fw*_ *2*.*5 cm*), soil temperature at 2.5 cm depth (*T*_*s*_ *2*.*5 cm*), soil temperature at 5 cm depth (*T*_*s*_ *5 cm*), and solar radiation (*SR*) during the different growth stages of the rice plants (vegetative, reproductive, and ripening) in 2013 and 2014 wet seasons (WS).

	2013WS						2014WS					
	vegetative		reproductive		ripening		vegetative		reproductive		ripening	
Variables	*r*	*ρ*	*r*	*ρ*	*r*	*ρ*	*r*	*ρ*	*r*	*ρ*	*r*	*ρ*
*Ta*	0.88	<0.0001	-0.17	0.2539	0.24	0.1015	0.86	<0.0001	0.67	<0.0001	0.45	0.0015
*T*_*fw*_ *2*.*5 cm*	0.87	<0.0001	-0.06	0.6652	0.35	0.0162	0.86	<0.0001	0.61	<0.0001	0.41	0.0034
*T*_*s*_ *2*.*5 cm*	0.90	<0.0001	0.18	0.2220	0.36	0.0133	0.89	<0.0001	0.78	<0.0001	0.54	<0.0001
*T*_*s*_ *5 cm*	0.83	<0.0001	0.32	0.0247	0.43	0.0023	0.78	<0.0001	0.78	<0.0001	0.54	<0.0001
*SR*	0.77	<0.0001	-0.35	0.0162	0.02	0.9027	0.95	<0.0001	0.53	0.0001	0.33	0.0230

We also accounted for the temporal variability of diurnal and nocturnal fluxes during each growth stage and expressed them as coefficient of variation (Table 4). Generally, the diurnal fluxes had higher variability than the nocturnal fluxes. Across two years, the diurnal fluxes had about (1.8 ± 0.8) times higher coefficients of variation than the nocturnal fluxes.

**Table 4:** Coefficient of variation (%) of diurnal and nocturnal CH_4_ fluxes during the different growth stages of the rice plants (vegetative, reproductive, and ripening) in 2013 and 2014 dry (DS) and wet seasons (WS).

	2013DS		2014DS	
Growth stage	Diurnal	Nocturnal	Diurnal	Nocturnal
Vegetative	51.3	26.4	32.9	13.5
Reproductive	30.2	10.0	18.9	16.1
Ripening	36.9	26.1	39.7	34.5
	2013WS		2014WS	
Growth stage	Diurnal	Nocturnal	Diurnal	Nocturnal
Vegetative	35.1	11.7	31.0	11.0
Reproductive	8.3	8.1	15.9	14.9
Ripening	22.7	22.9	32.6	29.3

#### Relationship of nocturnal fluxes with diel fluxes

**Fig 3:**
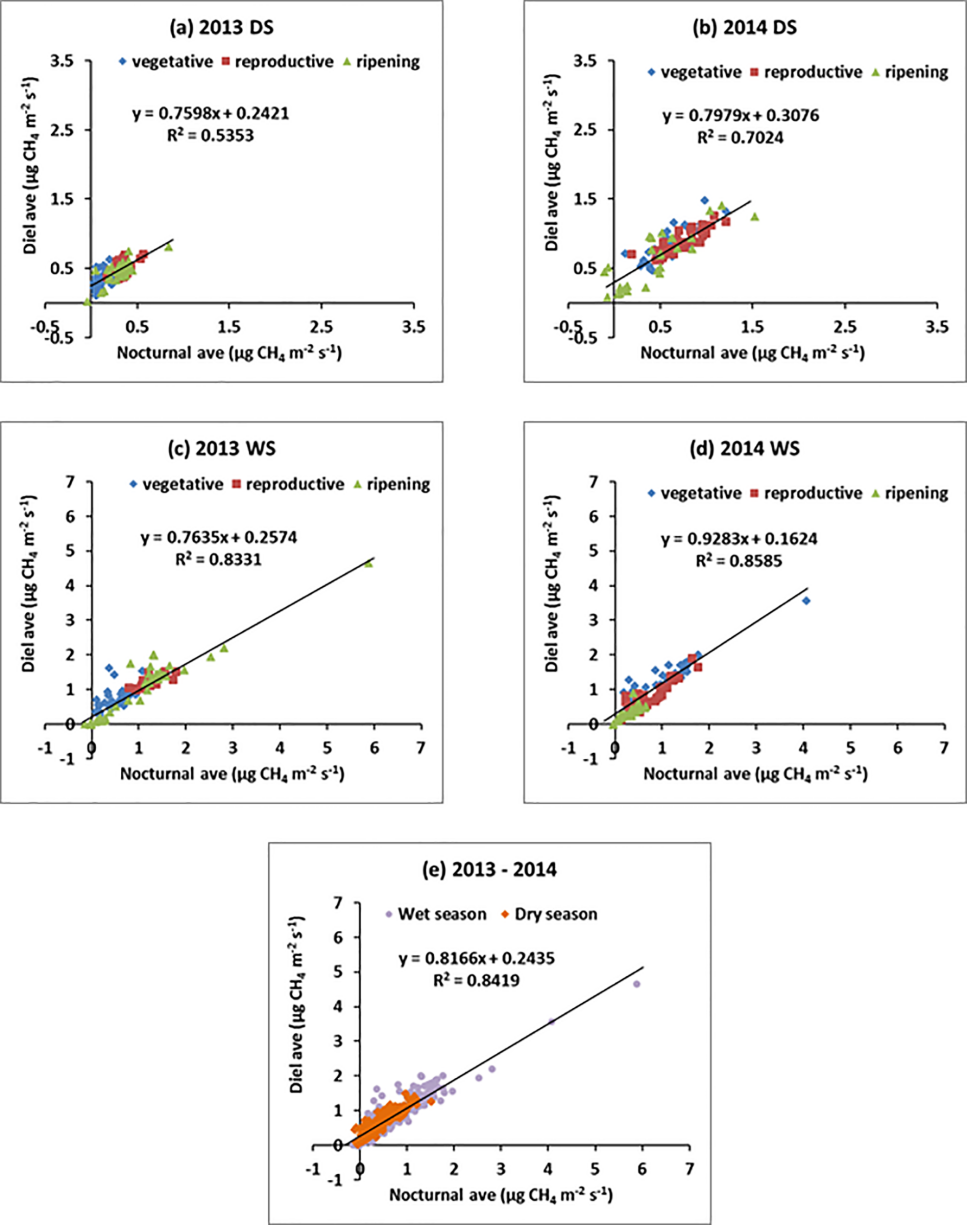
Linear relationship of nocturnal CH_4_ flux with diel CH_4_ flux during (a) 2013DS, (b) 2014DS, (c) 2013WS, (d) 2014WS, and (e) pooled relationship. DS–dry season and WS–wet season. See below.

Fig 3A–3D displays the averages of nocturnal and diel fluxes. The strong linear relationship is evident for all 4 seasons. During the dry seasons, nocturnal flux had significant and very strong positive relationship with the diel flux with r values ranging from 0.73 to 0.84 (Fig 3A and 3B). Linear regression analysis showed that nocturnal flux accounted for almost 54% of the variation in diel flux for 2013DS and 70% for 2014DS. During the wet seasons, the correlation between nocturnal and diel flux is even more pronounced with r values ranging from 0.91 to 0.93 (Fig 3C and 3D). Almost 83–86% of the variation in diel flux can be accounted for by the variation in nocturnal flux for 2013WS and 2014WS. Fig 3E shows the pooled linear relationship of nocturnal CH_4_ flux with diel CH_4_ flux with an R^2^ = 0.84 and RMSE = 0.20.

Both data sets of the wet seasons included one data point with high values for nocturnal and diel averages. As we recognize that such ‘outliers’ could potentially distort a statistical correlation analysis, we have also computed r values for both seasons by discarding the two data points. However, the correlation is still very strong with r values of 0.86 (2013WS) and 0.91 (2014WS).

### Experiment B: Nighttime closed-chamber measurements

There was only a slight decrease in temperature (3.3 ± 2.3°C) inside the chamber from 18:00H to 24:00H hours as compared to the temperature increase (8.9 ± 7.1°C) that we typically observe from 0900H to 1100H. Further, nighttime changes in oxygen concentrations inside the chamber were minimal at 0.14 ± 0.033% h^-1^ i.e. 21.0 to 20.2% from 1800H to 2400H.

**Fig 4:**
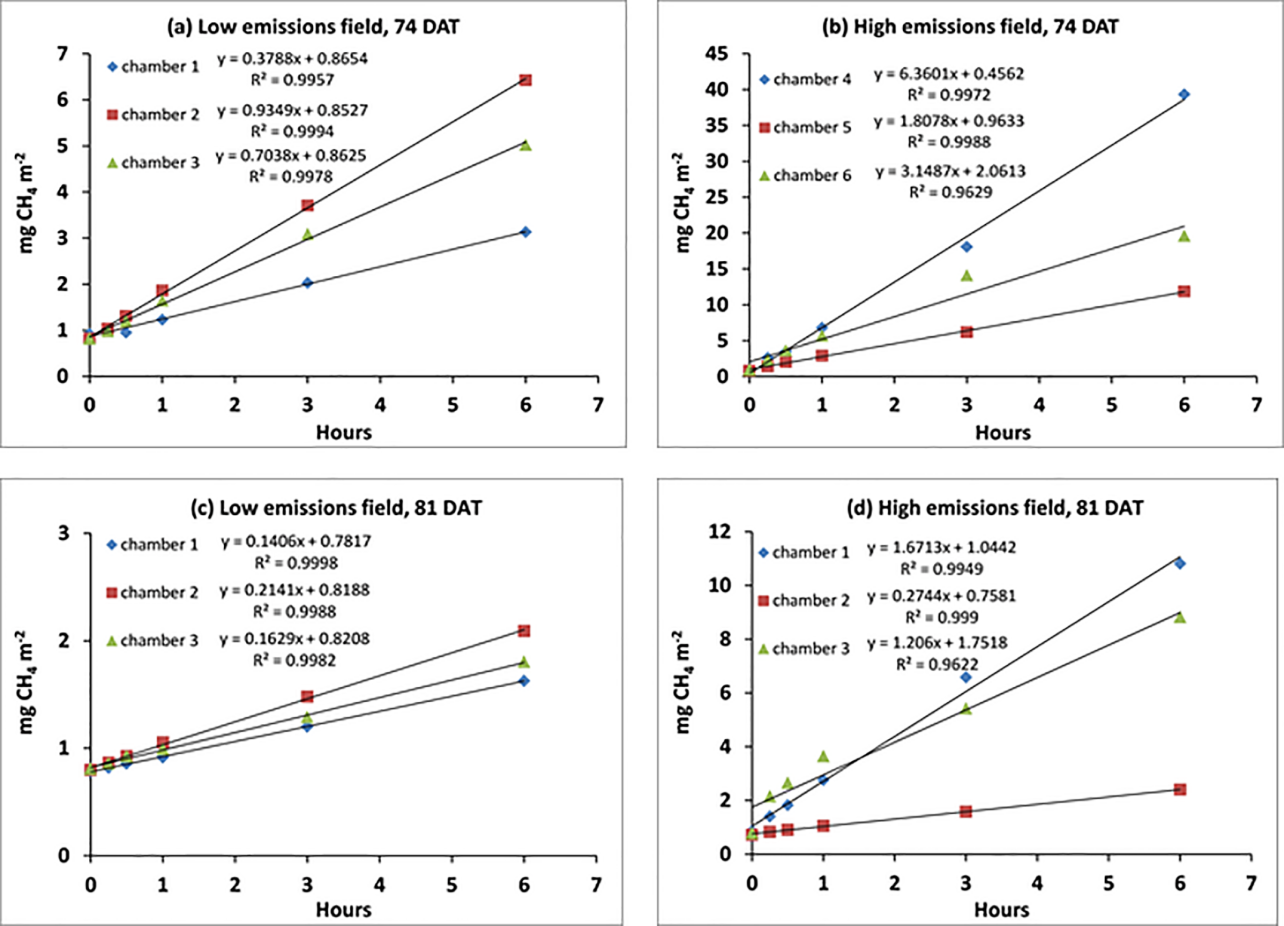
Linear increases in CH_4_ fluxes inside closed chambers in (a) a low emissions field at 74 DAT, (b) a high emissions field at 74 DAT, (c) a low emissions field at 81 DAT, and (d) a high emissions field at 81 DAT.

The measured increments in CH_4_ concentrations inside closed chambers between 18:00H and 24:00H hours consistently showed significant linear trends (R^2^ = 0.962–0.999, Fig 4). These measurements have been conducted during 74 and 81 DAT, corresponding to the early part of the ripening stage. The linearity was sustained up to six hours of chamber deployment even at high levels of CH_4_, which reached up to 88 ppm (Fig 4B). The slope of the regression line represented the concentration increase in ppm h^-1^. Longer enclosure periods (> 1 hour) led to much higher increments in CH_4_ emissions and the increase were all way above the LOD_gc_ of 0.3 ppm.

In Table 5, we show the concentration increments of the nighttime experiment with the lowest emission rates (see Fig 4C). Given the incumbent GC settings, there is no detection of fluxes possible for a short enclosure period of 30 min and in one chamber not even for 1h. However, the concentration increments of 3h and 6h deployment time are well above the required LOD_flux_ values.

**Table 5:** Incremental changes in CH_4_ concentrations and derived flux rates measured under low background level of CH_4_ fluxes; concentration increments below LOD_gc_ and LOD_flux_ are marked in red cells.

	Enclosure time (h)	Concentration (ppm)		Flux (mg m^-2^ h^-1^)	
		Increment (Δ)	LOD_gc_	Increment (Δ)	LOD_flux_
Chamber 1	0–0.5	0.143	0.3	0.143	0.274
	0–1	0.264		0.129	0.173
	0–3	0.878		0.139	0.046
	0–6	1.803		0.140	0.023
Chamber 2	0–0.5	0.272	0.3	0.256	0.278
	0–1	0.544		0.256	0.139
	0–3	1.448		0.227	0.046
	0–6	2.748		0.215	0.023
Chamber 3	0–0.5	0.252	0.3	0.239	0.278
	0–1	0.385		0.186	0.139
	0–3	1.008		0.161	0.046
	0–6	2.105		0.166	0.023

## Discussion

### Diel variations in CH_4_ fluxes and correlation with radiation and temperature

This study has investigated the temporal variations of the diel patterns of CH_4_ flux using the eddy covariance technique during the different growth stages of the rice plant, and has compared the variability between two cropping seasons (dry and wet) for two years. Most previous studies on diel patterns of CH_4_ fluxes in rice were conducted using closed-chamber technique [20–23] whereas few studies were based on Eddy Covariance measurements [24, 25]. In this study, distinct diel patterns of CH_4_ flux were observed when our data were partitioned into different periods since CH_4_ emissions vary through time during the cropping season. The temporal variations of the diel CH_4_ flux during the dry season were more pronounced than during the wet season because the latter had so much climatic disturbance from heavy monsoon rains and occasional typhoons.

In the dry season (across two years), the nocturnal CH_4_ fluxes started at a moderately high level but decreased rapidly until 1900H and remained at low levels until 0600H. In contrast, the diurnal fluxes increased with accelerating rates in the morning, reached a peak in the early afternoon, and then decreased steadily until nighttime. These patterns were similar to those reported by other studies [19–24]. Similar patterns were also observed during the wet seasons but only during the vegetative stage of the rice plant. The amplitude between the minimum and maximum fluxes was higher during the vegetative stage (across two years) with ratio of about 5.7 ± 3.9 compared to reproductive and ripening stages with ratio of 4.1 ± 2.6. Our values are similar to the diurnal amplitudes reported by other studies [9, 26, 27].

The CH_4_ flux patterns were correlated to diel patterns of *Ta*, *T*_*fw*_ *2*.*5 cm*, *T*_*s*_ *2*.*5 cm*, *T*_*s*_ *5 cm*, and *SR*. It has been well documented from previous studies that in most environments, the methanogens and methanotrophs become more active as solar radiation warms the soil, which enhances CH_4_ production and oxidation [28, 29]. Increase in soil temperature also increases (a) CH_4_ transport through the aerenchyma of the rice plant [30]; (b) molecular diffusion of CH_4_ [31]; and (c) ebullition of CH_4_ [32].

### Relationship of nocturnal fluxes with diel fluxes

Our results show that measurements during nighttime represent suitable approach for determining diel flux rates–irrespective of season or plant stage. Even during the wet season, the absence of diel patterns did not affect the strong correlation between nocturnal and diel averages. The linear relationships between the nocturnal and diel CH_4_ fluxes during the wet seasons (across two years) had even higher r values because there were no distinct diel patterns during the reproductive and ripening stages and so flux values were distributed randomly. In Table 6, we show two distinct emission ratios for the nocturnal averages for every season. Across 4 seasons, the nocturnal CH_4_ emissions were 20% lower than that of the CH_4_ emissions over the entire 24-h cycle while they were 21% lower than emissions during daylight (Table 6).

**Table 6:** Emission ratios depicting the relative share of nocturnal emissions derived from 30-min recording intervals over 4 seasons; emission records of a given day (diel period) were divided into nocturnal period (00:30–06:00 and 18:30–24:00) and diurnal period (06:30–18:00).

Cropping season	Ratio nocturnal vs diel	Ratio nocturnal vs diurnal
2013DS	0.60	0.43
2014DS	0.76	0.62
2013WS	0.96	0.92
2014WS	0.88	0.79
MEAN	0.80	0.69

### Proof of concept for chamber deployment at nighttime

The linear increase in CH_4_ concentrations within six hours of chamber deployment from 18:00H to 24:00H hours can be attributed to 2 causes that are conducive for developing a new measurement approach with nighttime chamber deployment:

- long enclosure intervals and high CH_4_ concentrations do not result in any saturation point for CH_4_ entering the chamber headspace
- emission rates are very stable during nighttime, so the measurement time are not biased by the respective time slot as during daylight

CH_4_ fluxes showed strong positive correlations with solar radiation and temperature over a given 24-h cycle (Table 2). This finding is tantamount to steady emission rates during nighttime when variations in solar radiation and temperature inside the chamber are at a minimum. The change in oxygen concentration was also minimal within the six-hour chamber deployment at nighttime that would unlikely affect microbial activities. The steady emission rates observed at nighttime provide proof that reliable estimates of CH_4_ fluxes could be obtained through longer chamber deployment at nighttime–at least up to 6-h periods as shown in this study, but possibly even for 12-h periods.

This finding should be seen in the context of striving for improved sensitivity in flux measurement. Eq 3 shows that an accurate GC analysis (LOQ_gc_) is obviously important for attaining low LOQ_flux_. Moreover LOD_flux_ can be reduced–or sensitivity be increased—by either (i) lower chamber height (as expressed by $\frac{V}{A}$) or (ii) longer deployment time. Using shallow chambers is constrained by the nature of CH_4_ transport to the atmosphere which is largely conveyed through the rice plants. Thus, chambers have to enclose fully developed plants without causing mechanical damage. Longer deployment time of the chambers will also cause damage to the plants–as long as measurements are done during daylight. At nighttime, however, longer deployment periods of the chambers have no adverse effects on the plants.

## Conclusions

The new approach of this study envisages nighttime measurements although flux rates are lower than during daylight. Derived from Table 6, the nocturnal emissions rates correspond to 80% of the average over a 24-h cycle and to 69% of the values recorded during a 12-h interval at daylight. In the first instance, the low emissions rates would speak against doing measurements at night. However, longer enclosure intervals easily compensate for the lower level of emission rates. During nighttime, enclosure times of the chambers can be expanded to several hours because temperature conditions inside the chamber are stable. In turn, this entails much higher end concentrations for CH_4_ and facilitates precise detection of flux rates. The enclosed air volume also experiences smaller concentration changes of O_2_ (decrease) and CO_2_ (increase), but those will not exert negative impacts on plant performance.

We acknowledge that the choice of methodology will always depend on the measurement objectives and that the ‘conventional’ approach with short enclosure times during daylight will remain the method of choice for many applications. At the same time, a modified methodology with nighttime deployment of chambers entails clear advantages for the following project objectives:

1. Detection of low emission baselines
2. Comparative assessments of crop management treatments that only have small differences in emissions
3. Comparative assessments of a large number of treatments or cultivars

While the 1^st^ and 2^nd^ objective have been addressed above in the discussion, the 3^rd^ objective deserves more elaboration. Identification of low emitting varieties has often been suggested as a promising approach for reducing CH_4_ emissions [33–36]. However, only few studies have addressed the different characteristics of rice cultivars that can reduce CH_4_ emissions against the backdrop of enormous genotypic and phenotypic variations amongst rice genotypes [37, 38]. The rice plant is involved in CH_4_ emissions through several pathways that may be exploited for mitigation, namely (i) root exudation providing substrate for methanogenic bacteria [39, 40]; (ii) gas transport capacity through the aerenchyma [34, 41]; (iii) capacity to partition photosynthate to the panicles at the expense of the roots [42, 43]; shorter plant types [44]; and high root oxidation potential [45, 46].

Once the decisive traits have been identified, plant breeding could lead to the generation of low-emitting rice varieties that also have a high yield potential. The combination of these traits could offer an economically feasible and environmentally sound breeding strategy with the aim of mitigating CH_4_ emissions from wetland rice fields. While rice breeding has a strong track record to improve rice production systems, this goal can also be seen in the context of rapidly developing genome sequencing programs such as the 3k Rice Genome Project [47, 48]. As complementary Genome-Wide Association Studies (GWAS) are screening for specific plant traits and responsible genes that can then, be incorporated into breeding programs. Such advanced bio-technology/ bio-statistics approaches have been proven to be highly successful to develop new rice varieties with improved resilience against drought and other climatic stresses. However, the nature of GWAS applications demands for high-throughput phenotyping of a minimum of 100 cultivars including the necessary replicates. In the case of rice, the 3k Rice Genome Project provides an enormous wealth of genetic information, but it will require innovative approaches of high-throughput phenotyping are required to take full advantage.

While sensor technology has been very successful for to detect specific traits of plant performance from large arrays of plants, sensor applications for recording atmospheric exchange have to cope with greater challenges as for recording morphological parameter of the plants. The atmospheric exchange of vegetation is determined by highly variable eddies, so that the sensor-based detection without chambers can only be captured by measurement approaches for larger areas, e.g. the EC technique requiring a minimum fetch of 100m. This approach needs a square area of 4 ha that is uniformly managed and planted with only one variety. It seems obvious that this large area requirement cannot be met for screening high numbers of rice varieties, so that the closed chamber technique is the method of choice for high-throughput phenotyping for emissions.

The results from this study can collectively be interpreted as proof of concept of the proposed modification on the ‘closed chamber’ approach, namely to determine methane emission rates based on nighttime enclosure intervals. In the next step, we will have to transform this proof of concept into actual measurement protocols that take full advantage of the higher sensitivity of the new approach.
